# *rpoS*-mutation variants are selected in *Pseudomonas aeruginosa* biofilms under imipenem pressure

**DOI:** 10.1186/s13578-021-00655-9

**Published:** 2021-07-21

**Authors:** Xiangke Duan, Yanrong Pan, Zhao Cai, Yumei Liu, Yingdan Zhang, Moxiao Liu, Yang Liu, Ke Wang, Lianhui Zhang, Liang Yang

**Affiliations:** 1 Guangdong Province Key Laboratory of Microbial Signals and Disease Control, Integrative Microbiology Research Center, South China Agricultural University, Guangzhou, 510642 Guangdong People’s Republic of China; 2 School of Medicine, Southern University of Science and Technology, Shenzhen, 518055 Guangdong People’s Republic of China; 3 Southern University of Science and Technology Hospital, Shenzhen, 518055 Guangdong People’s Republic of China; 4 Department of Pulmonary and Critical Care Medicine, The First Affiliated Hospital of Guangxi Medical University, Nanning, 530021 Guangxi People’s Republic of China; 5 Shenzhen Key Laboratory for Gene Regulation and Systems Biology, Southern University of Science and Technology, Shenzhen, 518055 Guangdong People’s Republic of China

**Keywords:** Experimental biofilm evolution, *Pseudomonas aeruginosa*, Sigma factor RpoS, Biofilms, Cyclic-di-GMP, Virulence

## Abstract

**Background:**

*Pseudomonas aeruginosa* is a notorious opportunistic pathogen causing various types of biofilm-related infections. Biofilm formation is a unique microbial strategy that allows *P. aeruginosa* to survive adverse conditions such as antibiotic treatment and human immune clearance.

**Results:**

In this study, we experimentally evolved *P. aeruginosa* PAO1 biofilms for cyclic treatment in the presence of high dose of imipenem, and enriched hyperbiofilm mutants within six cycles in two independent lineages. The competition assay showed that the evolved hyperbiofilm mutants can outcompete the ancestral strain within biofilms but not in planktonic cultures. Whole-genome sequencing analysis revealed the hyperbiofilm phenotype is caused by point mutations in *rpoS* gene in all independently evolved mutants and the same mutation was found in *P. aeruginosa* clinical isolates. We further showed that mutation in *rpoS* gene increased the intracellular c-di-GMP level by turning on the expression of the diguanylate cyclases. Mutation in *rpoS* increased pyocyanin production and virulence in hyperbiofilm variants.

**Conclusion:**

Here, our study revealed that antibiotic treatment of biofilm-related *P. aeruginosa* infections might induce a hyperbiofilm phenotype via *rpoS* mutation, which might partially explain antimicrobial treatment failure of many *P. aeruginosa* biofilm-related infections.

**Supplementary Information:**

The online version contains supplementary material available at 10.1186/s13578-021-00655-9.

## Introduction

Microbial cells undergo rapid evolution under stress to adapt to the environment in nature [1] or inside the host [2]. Certain key mutations on genome can greatly help bacterial populations to gain a competitive advantage in adverse environment [2]. To identify these adaptive traits of microbes, experimental evolution experiments are usually conducted to mimic these diverse environmental conditions for accelerating the emergence of well-fitted variants [3]. Employment of next-generation sequencing approaches will facilitate the identification of the mutations of experimental adapted bacterial variants and elucidation of the underlying molecular mechanism of the evolved traits [4]. So far, adaptive experimental evolution has been applied to reveal the molecular basis of drug resistance [5], persistence [6], biofilm formation [7] and etc. An important question for experimental evolution is the relevance of laboratory observation to the evolution in natural conditions. For this point, studies have indicated the mutation derived phenotypes in laboratory evolution could be found in clinical isolates [8–11].

Bacterial pathogens can form biofilms on both biotic and abiotic surfaces, causing many hospital-acquired and recurrent infections. Biofilms are densely-packed microbial cells embedded in self-secreted hydrated matrix consisting of polysaccharides, proteins, extracellular DNA and lipids [12]. Previous studies have shown that the biofilm-grown bacteria have distinct phenotypes from planktonic cultures, including gene expression [13, 14] and increased antibiotic resistance [15]. The tolerance to antimicrobial agents by biofilm cells can be increased up to 1000-fold compared to planktonic cells [16]. Investigating evolution traits of biofilm cells against antibiotics might provide knowledge about bacterial adaptation during chronic infections.

*P. aeruginosa* is a notorious opportunistic pathogen, which causes a variety of infections, including wounds, urinary tract and respiratory tract infections, and is the leading cause of morbidity and mortality for people suffering from cystic fibrosis (CF) [17]. Infections caused by *P. aeruginosa* can be very difficult to treat due to its intrinsic resistance to a variety of antibiotics and tends to form biofilms at the sites of infection [18, 19]. As an important nosocomial pathogen, *P. aeruginosa* biofilms have been found on various surfaces of indwelling medical devices, including urinary catheters, bone plates, ventricular assist device drivelines and pacemakers [20]. Biofilms provide *P. aeruginosa* an enormous advantage in clinical infections by protecting biofilm cells from the immune clearance [21] and tolerance to antimicrobial agents [22, 23]. Persisters are subpopulation of isogenic bacteria that tolerance to antibiotics [24] and the persister cells were dormant in biofilms, which significantly contributes to *P. aeruginosa* biofilm recalcitrance after the cessation of antibiotic therapy [25]. Considering the issue of drug tolerance and recalcitrance of *P. aeruginosa* biofilm-related infections, novel anti-biofilm therapeutics are urgently needed.

**Fig. 1:**
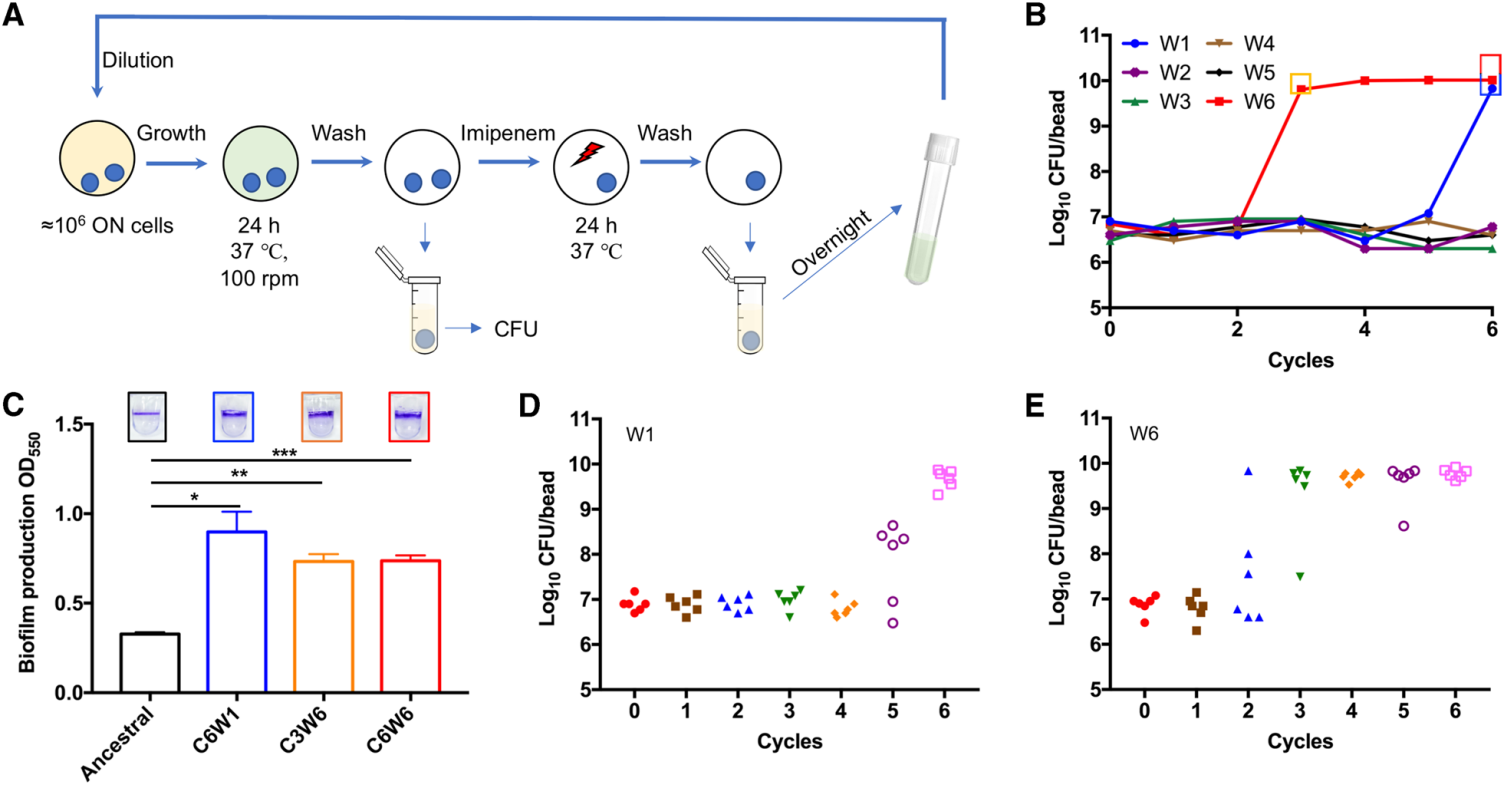
Experimental biofilm evolution of *P. aeruginosa* under antibiotic stress. **A** The setup of experimental biofilm evolution of *P. aeruginosa*. The biofilm of six independent lineages of the *P. aeruginosa* PAO1 were grown on 5 mm glass bead. After 24 h cultivation, one bead was vortex and sonicated for CFU counts, another bead was transferred to a 24 well microplate and treated with 160 μg/mL imipenem. After 24 h treatment, the surviving cells were grown overnight in fresh medium and start another cycle. **B** Evolution of biofilm bacteria exposed to imipenem resulted in a rapid increase in biofilm bacteria CFU on bead. **C** Crystal violet (CV) staining of biofilms formed by ancestral and hyperbiofilm variant strains on PVC plate. Data are presented as the mean ± s.d. of five biological replicates. Significance was determined using a Student’s *t* test: *P < 0.05, **P < 0.01 and ***P < 0.001. **D** and **E** The time frame of emergence of hyperbiofilm variants in linage W1 (**D**) and W6 (**E**). The biofilm formation by the different colonies was displayed with CFU of biofilm cells on 5 mm glass bead

The long-term use of antibiotics in the treatment of *P. aeruginosa* infections in cystic fibrosis patients is well-known to drive emergence of diversified drug-resistant variants. Clinical isolates of *P. aeruginosa* have shown distinct biofilm formation capacity and many of them were strong biofilm producers [26]. However, current adaptive experimental evolution studies of *P. aeruginosa* are mainly focusing on planktonic cultures [10, 27, 28]. Carbapenems, including Imipenem, were considered as the last resort of drugs for the treatment of multi drug resistant *P. aeruginosa* infections. The emergence of resistance to carbapenems limits its use for treatment [29]. In this study, we established an experimental evolution model to investigate the evolution traits of imipenem-treated biofilms of *P. aeruginosa*, and the set-up of this model is presented in Fig. 1A.

We show here that cyclic exposure of *P. aeruginosa* biofilms to high concentration of imipenem led to emergence of variants with a hyperbiofilm phenotype. The competition assay showed that the evolved hyperbiofilm variants can outcompete ancestral strain within biofilms but not in planktonic cultures. Genome sequencing analysis revealed that the hyperbiofilm phenotype is caused by single-point mutations in the sigma factor RpoS. Importantly, the mutations on *rpoS* identified in the in vitro experimental biofilm model also occurred in *P. aeruginosa* clinic isolates with a hyperbiofilm phenotype at a substantial rate. Overall, our data show that under imipenem treatment, mutations in *rpoS* could be selected in *P. aeruginosa* and subsequently lead to enhanced biofilm formation.

## Results

### Experimental biofilm evolution selects for *P. aeruginosa* hyperbiofilm mutants

In order to examine the evolutionary traits of *P. aeruginosa* biofilms under antibiotic stress condition, we exposed biofilms of *P. aeruginosa* PAO1 to different concentrations of imipenem (40, 80 and 160 μg/mL), which is 10, 20 and 40 times of the minimum inhibitory concentration (MIC), in a cyclic manner (Fig. 1). Imipenem, a widely used last resort antibiotic, has been chosen as the selective pressure for experimental biofilm evolution owing to its’ commonly prescribed for treatment of *P. aeruginosa* infections [30].

The biofilms of six independent lineages initiated from a common ancestor PAO1 strain were formed on the surface of 5 mm glass beads [31] and treated with different concentrations of imipenem for 24 h. Survivor cells on beads were quantitated by CFU counts. Biofilm survivors were collected and reinoculated in fresh LB medium for the 2nd cycle (Fig. 1A). At the first cycle, the CFU counts on bead of each lineage were between 6.30 and 6.90 log_10_. After 6 cycles, no hyperbiofilm variant was observed in control group or 10 × and 20 × MIC (Additional file 1: Figure S1A–C) imipenem treated groups, and two lineages treated by 40 × MIC of imipenem accumulated hyperbiofilm variants (Additional file 1: Figure S1D). The CFU counts within lineage W1 and W6 biofilms on bead reached 9.82 and 10.01 log_10_ in 40 × MIC imipenem treated group (Fig. 1B). Next, the biofilm formation capacity of the ancestral, C6W1, C3W6 and C6W6 (C refer to the cycle number, W refer to the linage number) population were further confirmed by the crystal violet (CV) biofilm assay. Similarly, the CV method revealed that biofilms formed by C6W1, C3W6 and C6W6 population were between 2- and threefold higher than the ancestral population (Fig. 1C). In order to track when the hyperbiofilm variants have emerged within lineage W1 and W6 population, we picked 6 colonies form each cycle in random and measured the CFU of biofilms on bead. We found that the hyperbiofilm variants of lineage W1 and W6 appeared since cycle 5 and cycle 2 and enriched at cycle 6 (Fig. 1D) and cycle 3 (Fig. 1E), respectively. These results indicate that the hyperbiofilm variants only accumulated upon higher concentration imipenem treatment, rather than lower concentration of imipenem. The different appearance time of hyperbiofilm variants in the two independent linages is possibly due to the variation of evolutionary rate [32]. More lineages might accumulate hyperbiofilm variants in 40 × MIC imipenem treated group if we increase the treatment cycles.

### Point mutations in *rpoS *lead to hyperbiofilm phenotype of *P. aeruginosa*

In order to elucidate the genetic mechanisms underlying the hyperbiofilm phenotype, we sequenced the C6W1 and C6W6 population, and choose C6W5 population as the negative control. Through comparative genomic analysis, we identified only SNPs in one gene, *PA3622* (encodes sigma factor RpoS) (Table 1), which was mutated in C6W1 and C6W6 population, and in contrast, no non-synonymous SNP was identified in C6W5 population when compared to the ancestral strain. The sigma factor RpoS is well known as a master regulator that controls the expression of genes involved in stress response and virulence factors production in *P. aeruginosa* [33, 34]. A previous study used transcript profiling and found that 772 genes were regulated by RpoS in stationary phase and it affects expression of more than 40% quorum-sensing controlled genes [35].

**Table 1:** Whole genome sequencing data for evolved *P. aeruginosa* populations

Strains	Region	Mutation	Mutation effect	Gene	Annotation
C6W1C	794674..794675	AC to GT	D89G	*PA0727*	Pf replication initiator protein
	4058162	G to A	P251L	*rpoS*	RNA polymerase sigma factor
C3W6F	794674..794675	AC to GT	G89G	*PA0727*	Pf replication initiator protein
	4058118	G to A	Q266Stop	*rpoS*	RNA polymerase sigma factor
C6W6F	4058118	G to A	Q266Stop	*rpoS*	RNA polymerase sigma factor

**Fig. 2:**
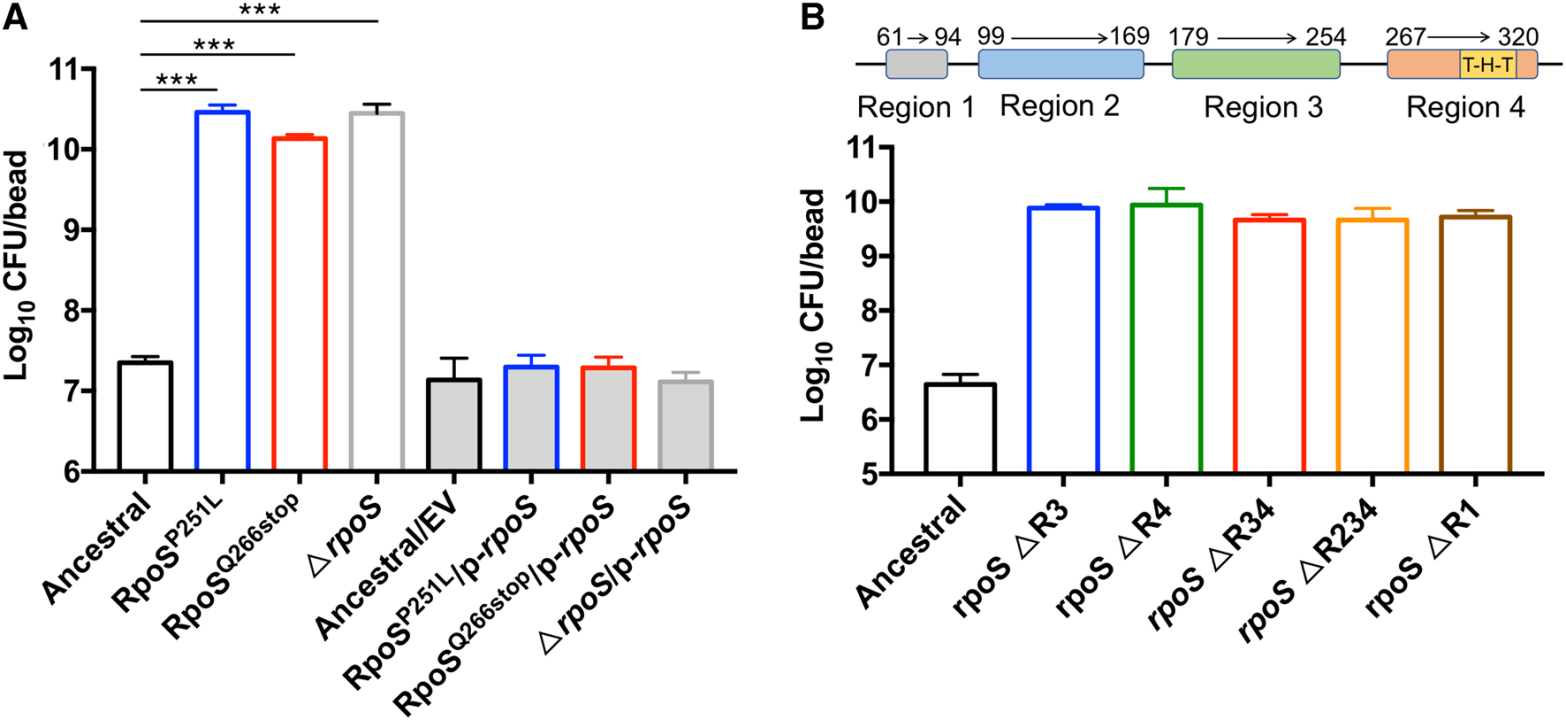
Hyperbiofilm phenotype is caused by *rpoS* mutation. *rpoS* point mutation, full deletion (**A**) and regions deletion strains (**B**) were increased the biofilm formation. The biofilm formation by the indicated strains was displayed with CFU of biofilm cells on 5 mm glass bead. Data are presented as the mean ± s.d. of four biological replicates. Significance was determined using a Student’s *t* test: ***P < 0.001. EV represents the empty vector pHERD20T in this assay

**Fig. 3:**
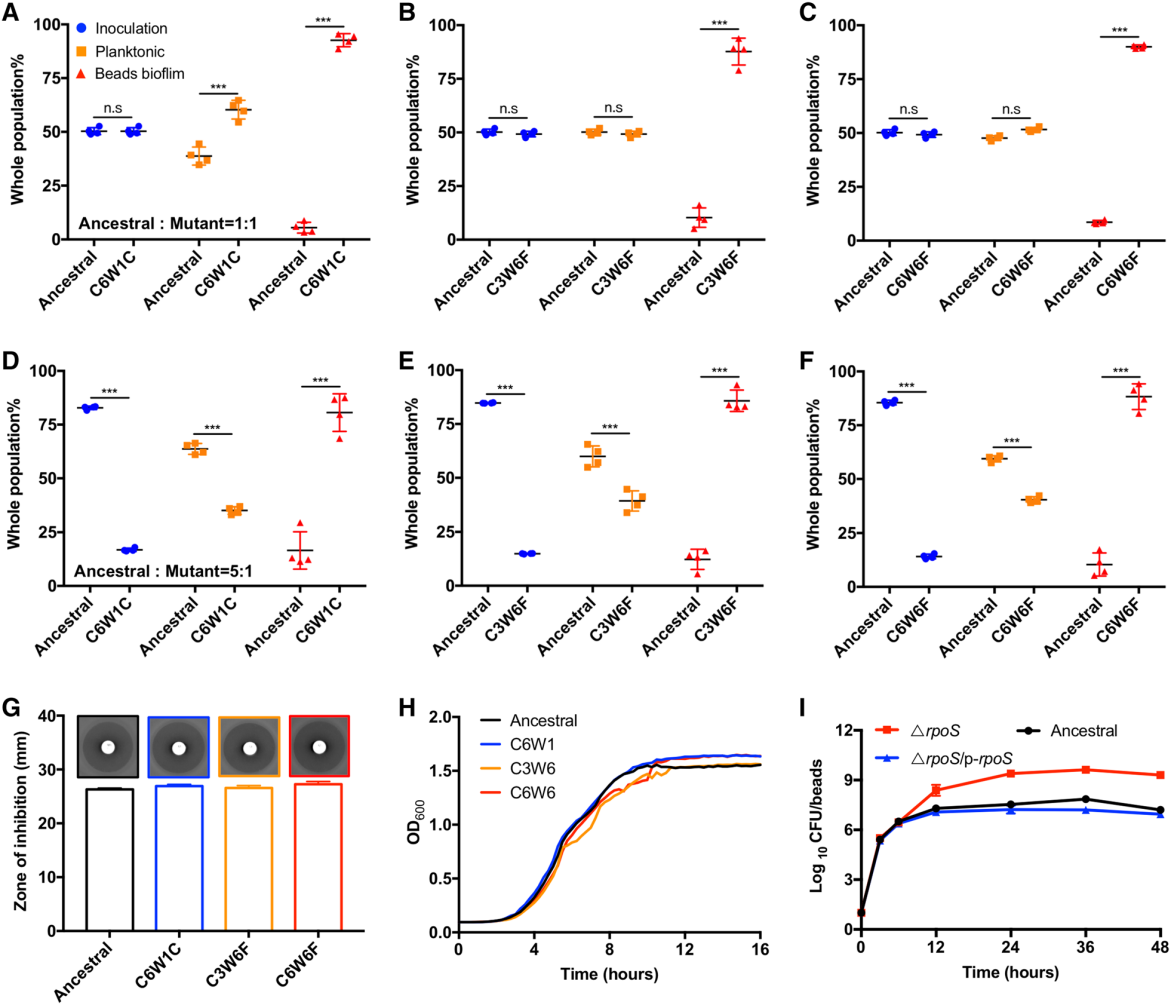
Hyperbiofilm variants prevail in biofilm competitions. **A**–**F** The competition of ancestor and hyperbiofilm variants in planktonic cultures and biofilms when inoculated at the same ratio (**A**–**C**) or ancestral: hyperbiofilm mutant = 5:1 (**D**–**F**). **G** Disc diffusion antibiotic sensitivity testing, **H** growth curves measurement, and **I** biofilm growth curve was measured. Data are presented as the mean ± s.d. of four biological replicates. Significance was determined using a Student’s *t* test: n.s indicates no significant difference (*P* ≥ 0.05); ***P < 0.001

Targeted resequencing of *rpoS* in C6W1C, C3W6F and C6W6F (colonies isolated from C6W1, C3W6 and C6W6 population) identified the nonsynonymous mutations in RpoS of C6W1C (P251L), C3W6F (Q266stop) and C6W6F (Q266stop). To further confirm the causality of *rpoS* mutations for the hyperbiofilm phenotype, we constructed a de novo mutant allele with a SNP on the ancestor PAO1 genome to yielded RpoS^P251L^ and RpoS^Q266stop^ mutant strains. We found that single point mutation in *rpoS* could produce the hyperbiofilm phenotype (Fig. 2A). We also tested the biofilm formation capacity of Δ*rpoS* strain and confirmed that knockout *rpoS* in *P. aeruginosa* PAO1 indeed increased the biofilm formation (Fig. 3A). Complementing the mutation strains with wild type *rpoS* reverted the hyperbiofilm phenotype to the wild-type level (Fig. 2A).

Protein domain analysis showed that RpoS consists of 4 regions, and region 4 contains a DNA binding domain. P251L located on the end of region 3, and knockout this region increased the biofilm formation. Q266stop mutation leads to RpoS lacking region 4, and knockout region 4 has the same phenotype of Q266stop mutation. Moreover, we have constructed region 1, region 3–4 and region 2–4 deletion strains, all of those region deletion mutants produced the hyperbiofilm phenotype (Fig. 2B). Together, these results provide a solid evidence that the SNPs identified are necessary and sufficient to cause hyperbiofilm phenotype in *P. aeruginosa*. Moreover, P251L and Q266stop mutations are very likely lead to the inactivation of RpoS.

### Hyperbiofilm variants outcompete the ancestral strain during biofilm competitions

The convergent emergency of *P. aeruginosa* hyperbiofilm variants from independent lineages suggested a competitive advantage for these variants over the ancestor. We have previously showed that *P. aeruginosa* cells did not share its EPS with its neighboring cells [36] and thus we hypothesized that the evolved hyperbiofilm variants only gain advantage to the ancestor strain during biofilm mode of growth. We then tested the competition of isolated hyperbiofilm variants with the ancestral strain in both planktonic cultures and biofilms. The fluorescently tagged hyperbiofilm variants (tagged with mCherry) were mixed with ancestral strain (tagged with GFP) in different ratio and inoculated into the bead-containing 24 well microplates. After 24 h cultivation, the planktonic and biofilm cells were analyzed using flow cytometry. We found that, the proportion of planktonic cells of C6W1C was slightly higher than ancestral strain when inoculated at the same ratio (Fig. 3A), while no difference was found between C3W6F and C6W6F with ancestral strain when inoculated at the same ratio (Fig. 3B, C). Interestingly, the competitions in biofilms confirmed that the hyperbiofilm variants have a significant and predominant selective advantage against the ancestral strain (Fig. 3A–C). Next, we increased the inoculation ratio of ancestor and hyperbiofilm variants to 5:1, the proportion of ancestor within biofilm was still much less than hyperbiofilm variants (Fig. 3D–F). These results indicated that the hyperbiofilm variants increased their fitness against the ancestor strain only in biofilms and this phenotype is not related to the change of growth rate, drug resistance and tolerance. Moreover, the competition advantage of hyperbiofilm variants is growth model specific and did not occur in planktonic culture.

Previous studies showed that acquired mutations conferring beneficial traits such as antibiotic resistance will dominate the populations when exposing biofilm bacteria to high concentrations of antibiotic [37]. Therefore, the enrichment of the hyperbiofilm *rpoS* mutation variants could have been achieved due to their enhanced resistance to imipenem. We found, however, that the MIC of imipenem for colonies isolated from the evolved lines (C6W1C, C3W6F and C6W6F) was indistinguishable from that for their ancestor (Fig. 3G). One of the most straightforward ways to gain a competitive advantage is increasing the growth rate. To test this point, we measured the growth rates of the evolved hyperbiofilm variants and the ancestor in LB medium and found there is no significant difference between the hyperbiofilm variants and ancestor (Fig. 3H). Next, we measured the biofilm growth curve of PAO1, Δ*rpoS* and the complementation strain Δ*rpoS/*p*-rpoS* and confirmed that Δ*rpoS* mutant formed more biofilms than the PAO1 wild type at all stages of biofilm formation (Fig. 3I). These results indicated that it is not the resistance level and planktonic growth rate select Δ*rpoS* mutant.

### Mutations in *rpoS* lead to an elevated intracellular c-di-GMP levels

**Fig. 4:**
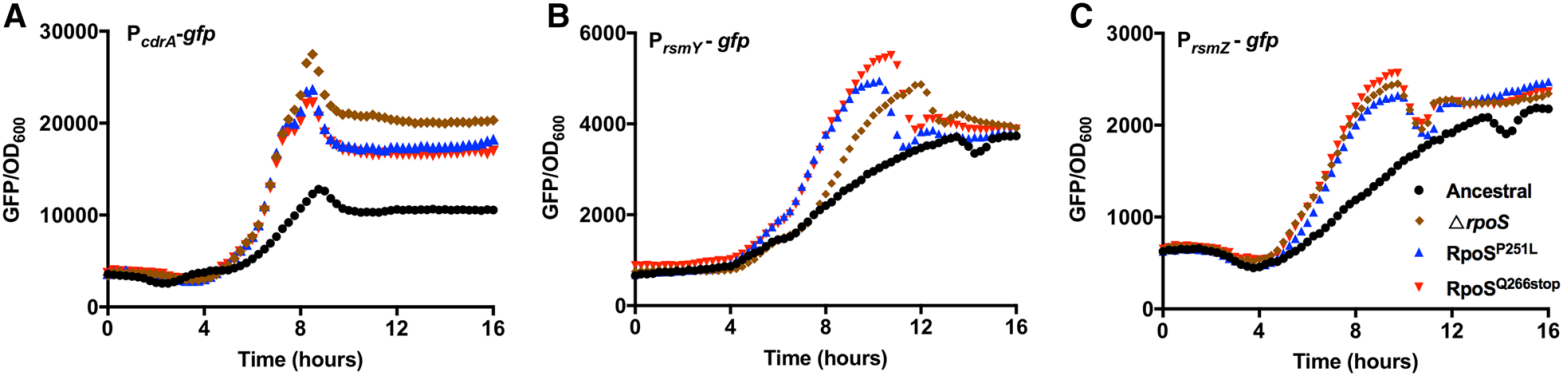
Expression of P_*cdrA-gfp*_, P_*rsmY-gfp*_ and P_*rsmZ-gfp*_ reporter fusions in *rpoS* mutants and PAO1 wild-type strain. Relative fluorescence intensity (reflected as GFP/OD_600_) was measured in representative strains containing the P_*cdrA*_-*gfp* (**A**), *rsmY* (**B**) and *rsmZ* (**C**) reporter fusions. Data are presented as the mean ± s.d. of five biological replicates

Quorum-sensing (QS) [38] and c-di-GMP [39] have been well documented to play important roles in regulating *P. aeruginosa* biofilm formation. To assess whether quorum-sensing and c-di-GMP levels were changed in the Δ*rpoS* variants, we introduced the quorum-sensing and c-di-GMP reporter systems [40–43] into the variants isolated from biofilm evolution experiments and ancestral strain to determine the relative levels of the corresponding signaling pathways. We found that the fluorescent signal of P_*lasB*_-*gfp* and P_*rhlA*_-*gfp* in ancestral strain were higher than that of hyperbiofilm variants (Additional file 1: Figure S2A and B), while there was no difference in fluorescent signal of P_*pqsA*_-*gfp* between the ancestral and hyperbiofilm variants (Additional file 1: Figure S2C). For the fluorescent signal of P_*cdrA*_-*gfp*, the hyperbiofilm variants showed twofold higher in expression level than the ancestral strain, indicating that the hyperbiofilm variants might have elevated intracellular c-di-GMP levels (Figure S2D). We further showed that the P_*cdrA*_-*gfp* expression level were increased in RpoS^P251L^, RpoS^Q266stop^ and Δ*rpoS* strain compared to the PAO1 wild-type strain (Fig. 4A). The second messenger c-di-GMP is a key regulator of *P. aeruginosa* biofilm formation, which is synthesized from two GTP molecules by diguanylate cyclases (DGC) and is degraded into 5ʹ-phosphoguanylyl-(3ʹ–5ʹ) guanosine (pGpG) and/or GMP by phosphodiesterases (PDE) [39]. Till now, 43 DGC and PDE proteins have been identified in *P. aeruginosa* [44].

To further investigate the regulatory roles of *rpoS* gene on c-di-GMP signaling pathway, we performed transcriptomic analysis of PAO1, RpoS^P251L^ and RpoS^Q266stop^ strains using RNA-sequencing. Samples were collected after 8.5 h culture owing to the P_*cdrA*_-*gfp* fluorescent intensity (Additional file 1: Figure S3) between mutants and wild type PAO1 strain have the biggest difference at this time point. We found that, 15 DGC and PDE encoding genes were upregulated by at least twofold in both RpoS^P251L^ and RpoS^Q266stop^ strains compared to PAO1 (Table 2). This result indicated that the c-di-GMP metabolism in *rpoS* mutant strains were more active than PAO1.

**Table 2:** The expression of GGDEF, EAL, HD-GYP proteins in *rpoS* mutation and ancestral strains

ORF	Name	Domain signature	Activity*	RpoS^P251L^ vs Ancestral FC	*p* value	RpoS^Q266stop^ vs Ancestral FC	*p* value
PA0169	*siaD*	GGEEF	DGC	− 2.17	3.39E-10	− 2.21	2.09E-10
PA2572		YN-GYP	–	7.12	5.02E-202	19.45	1.16E-234
PA0847		GGDEF	DGC	2.94	7.01E-42	3.09	4.48E-43
PA1107	*roeA*	GGEEF	DGC	2.16	3.15E-18	2.52	3.39E-23
PA1120	*yfiN*,	GGDEF	DGC	2.27	1.85E-19	2.85	2.19E-27
PA3343		GGEEF	DGC	–	–	2.09	1.07E-29
PA3702	*wspR*	GGEEF	DGC	2.17	5.79E-25	2.55	3.33E-34
PA4929	*nicD*	GGDEF	DGC	5.4	1.61E-174	8.85	2.60E-236
PA0290		GGEEF	ND	–	–	2	3.72E-17
PA0575	*rmcA*	GGDEF, EAL	ND	3.5	4.78E-92	4.63	3.54E-125
PA1181		GGDEF, ELL	ND	3.18	3.48E-79	3.56	3.54E-125
PA2771		GGEEF	ND	3.4	2.17E-60	4.25	4.65E-74
PA0861	*rbdA*	GGDEF, ELL	PDE	4.19	2.22E-140	6.93	7.11E-230
PA2072		GGDEF, EAL	PDE	7.55	8.41E-222	14.62	6.89E-293
PA3311	*nbdA*	AGDEF, EAL	PDE	7.65	6.06E-252	13.56	1.66E-279
PA3825		EVL	PDE	3	9.51E-46	3.45	9.00E-52
PA4108		HD-GYP	PDE	3.42	2.03E-80	3.9	2.38E-92
PA4781		HD-GYP	PDE	8.8	2.99E-306	14.18	2.78E-130

*ND not determined, – not active, *FC* fold change

RpoS regulates the expression of small regulatory RNAs *rsmY* and *rsmZ* in *Legionella pneumophila* [45]. Moreover, *rsmY/Z* participate in the regulation of c-di-GMP production in *P. aeruginosa*, the c-di-GMP levels were strongly reduced in the *rsmY*/*Z* double deletion mutant [46]. Our transcriptomic analysis showed that the expression of *rsmY* and *rsmZ* was increased 3.84 and 5.04-fold in RpoS^P251L^ compared to the PAO1 wild-type, respectively. Next, we measured the expression of *rsmY*/*Z* in PAO1, RpoS^P251L^, RpoS^Q266stop^ and Δ*rpoS* strains using reporter fusions [13]. We found the *rsmY*/*Z* expressions were increased in RpoS^P251L^, RpoS^Q266stop^ and Δ*rpoS* (Fig. 4B, C), which is consistent with the increased level of c-di-GMP of these mutants. These results showed that the mutation of *rpoS* has led to the increase in *rsmY*/*Z* expression and intracellular c-di-GMP content in *P. aeruginosa*.

### *rpoS* mutation associated hyperbiofilm phenotype in clinical isolates

**Fig. 5:**
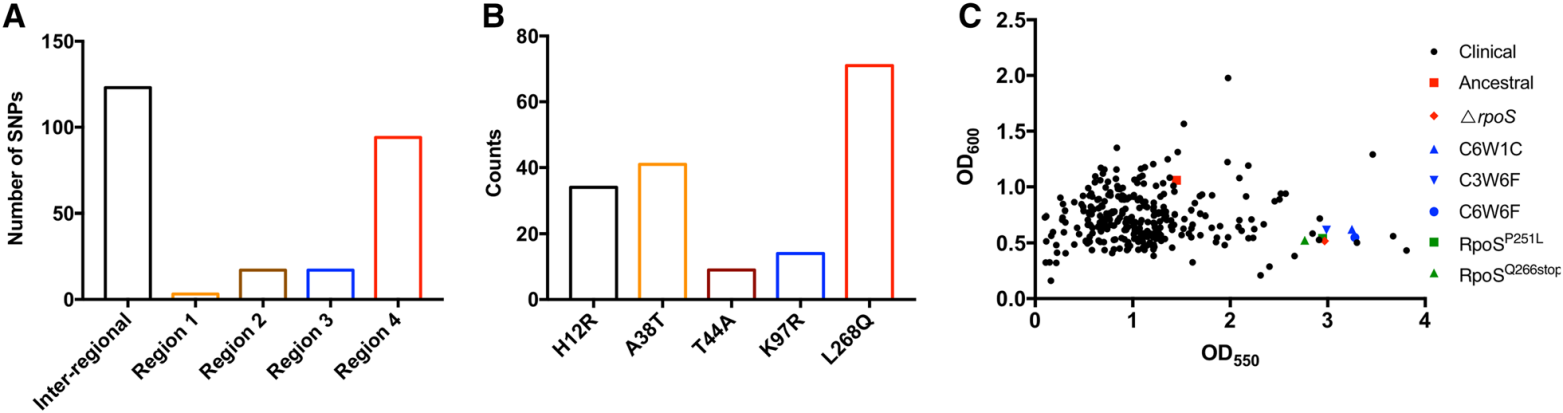
*rpoS* mutations were existed in *P. aeruginosa* clinical isolates. The distribution of non-synonymous mutation **A** and the top 5 mutations **B** on RpoS of *P. aeruginosa*. 4000 sequences of *rpoS* were download form pseudomonas genome database, and the non-synonymous mutations were analyzed by CLC Genomics Workbench. **C** Biofilm formation ability assessment of clinical isolates. A total of 288 clinical isolates from the first affiliated hospital of Guangxi Medical University (shown in black) were analyzed for *rpoS* mutations and biofilm assays on 96 well PVC plates

Our experimental biofilm evolution data has revealed that single-nucleotide mutations in *rpoS* confer *P. aeruginosa* hyperbiofilm phenotype and produce a pronounced competitive advantage within the biofilm microenvironment. In order to analyze the preference of *rpoS* mutation, we downloaded 4000 sequences of *rpoS* from the pseudomonas genome database (www.pseudomonas.com). Through comparative analysis, we have identified 241 non-synonymous mutations (6.03% of total sequence), 8 insertion or deletion mutations (0.2% of total sequence) and 5 stop coding mutations (0.13% of total sequence) compared to the PAO1 wild-type strain. Among those mutations, 123 mutations were in the sequence between annotated regions (inter-region) of RpoS and 131 mutations were within 4 regions (Fig. 5A). Moreover, we have identified 2 sequences harbored RpoS^P251L^ mutation. We also analyzed the top 5 mutation sites among the 4000 sequences, and found L268Q was the top one with 71 sequences (Fig. 5B).

Since imipenem has been used for clinical treatment of *P. aeruginosa* infections, we wondered whether *rpoS* mutation caused hyperbiofilm strains exist in clinical isolates. Therefore, we examined the biofilm formation capacity of 288 clinical *P. aeruginosa* isolates obtained from the patients with culture confirmed *P. aeruginosa* infections (Additional file 1: Table S1). Through quantitative analysis of biofilm formation by measuring crystal violet staining at OD_550_ nm and total bacterial growth at OD_600_ nm to exclude growth variation, we identified 29 hyperbiofilm isolates (10.07% of total isolates) in this collection (Fig. 5C). Next, we target sequenced *rpoS* of the 29 hyperbiofilm isolates and confirmed that #16 isolate harbored non-synonymous mutation in *rpoS*. Interestingly, #16 isolate, which is isolated from the peritoneal drainage fluid, has the same mutation RpoS^P251L^ as our experimental evolved variant C6W6F.

### The evolved *rpoS* variants are hypervirulent against host cells

**Fig. 6:**
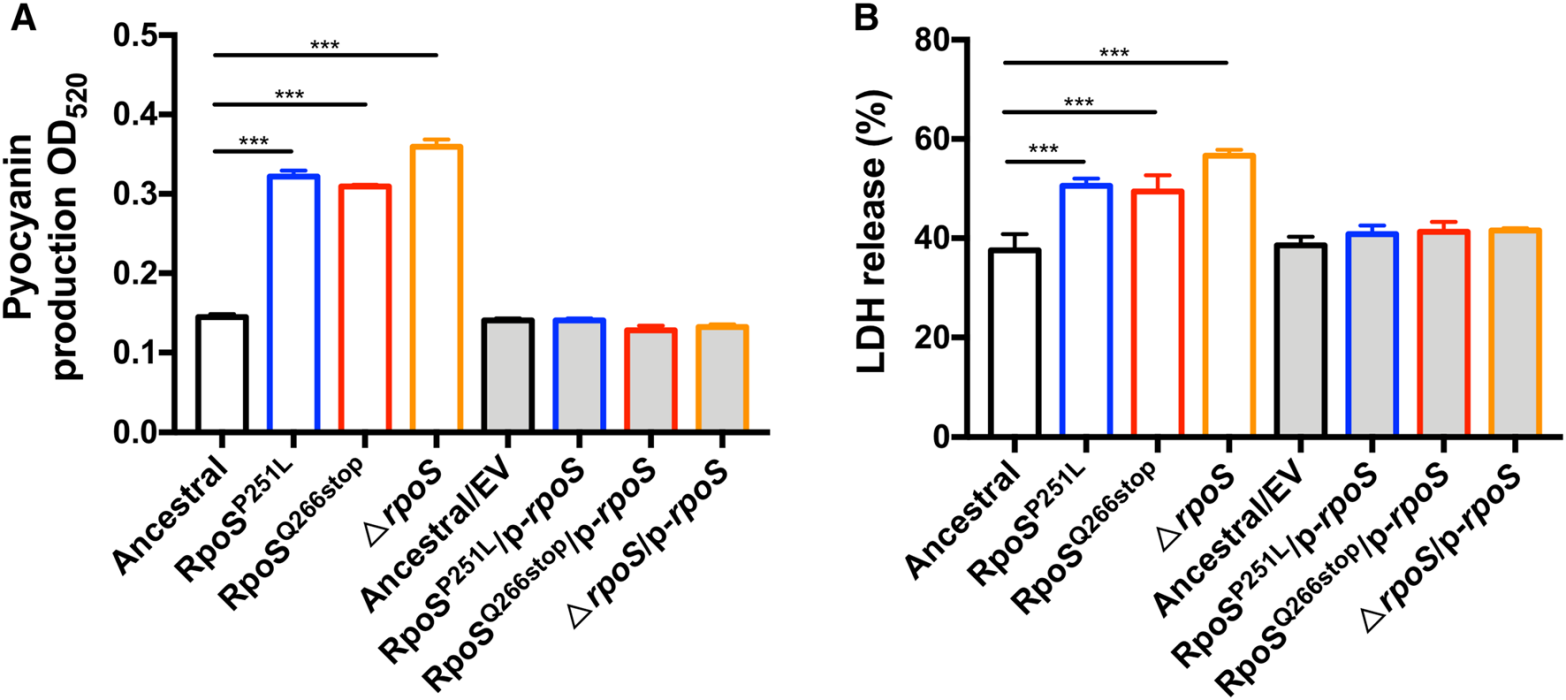
Pyocyanin production and virulence are increased in *rpoS* mutants. The production of pyocyanin (**A**) and cytotoxicity effect against macrophage cells (**B**) of *P. aeruginosa* PAO1 wild-type, RpoS^P251L^, RpoS^Q266stop^ and Δ*rpoS*. Data are presented as the mean ± s.d. of four biological replicates. Significance was determined using a Student’s *t* test: *P < 0.05, **P < 0.01, ***P < 0.001

Pyocyanin is one of the major virulence factors of *P. aeruginosa*, which causes oxidative stress to the host cells and induces apoptosis in neutrophils and inhibits phagocytosis of macrophages [47, 48]. Previous studies showed that the pyocyanin production was increased in a *rpoS*-deletion mutant [34]. To test the impact of *rpoS* point mutations of the biofilm evolved variants on pyocyanin production, we compared the production of pyocyanin by *P. aeruginosa* PAO1 strain, RpoS^P251L^, RpoS^Q266stop^ and Δ*rpoS*. As we expected, similar to the Δ*rpoS* mutant, the *P. aeruginosa* RpoS^P251L^ and RpoS^Q266stop^ produced higher amounts of pyocyanin than the wild-type PAO1 strain (Fig. 6A). This result suggests that point mutations accumulated in the *rpoS* gene in *P. aeruginosa* have similar effect as *rpoS* gene deletion on its physiology.

Next, we further assessed the impact of evolved *rpoS* point mutations on virulence using the macrophage cytotoxicity model [49]. The RAW264.7 macrophages were infected with *P. aeruginosa* PAO1, RpoS^P251L^, RpoS^Q266stop^ and Δ*rpoS*, and the release of cytosolic lactate dehydrogenase (LDH) was determined. We found that macrophages infected with RpoS^P251L^, RpoS^Q266stop^ and Δ*rpoS* released more LDH compared to *P. aeruginosa* PAO1 after 4 h infection (Fig. 6B). Altogether, these results suggest that mutations in *rpoS* can enhance the virulence in *P. aeruginosa*.

## Discussion

Biofilms represent the predominant lifestyle for most microorganisms in nature. Understanding how microorganisms evolve in biofilms can reveal novel insights of adaptive evolution, especially under stress conditions. For example, small colony variants are enriched in *P. aeruginosa* biofilms after exposure to sodium dodecyl sulfate [50]. The *P. aeruginosa* hyperbiofilm forming variants are often observed from patients who are suffering chronic infections such as CF [51]. We have previously using a planktonic experimental evolution model to demonstrate that oxidative stress drives the evolution *P. aeruginosa* hyperbiofilm variants carrying point mutations in the *wspF* gene, which lead to increase in intracellular c-di-GMP content and exopolysaccharide synthesis [27]. Here, we performed biofilm experimental evolution to examine the adaptive evolution of *P. aeruginosa* biofilms under the treatment of imipenem (160 μg/mL), which is a case can be encountered in clinical settings. The keratitis infection caused by *P. aeruginosa* usually form corneal biofilms [52]. In a case of the treatment of bacterial keratitis in patients, topical imipenem (50 mg/mL) has been selected as monotherapy for corneal infection [53]. In another study, 1–5 mg/mL imipenem has been used for bacterial keratitis treatment via topical administration [54]. Moreover, we showed that the hyperbiofilm variants could be accumulated in biofilms after cycle antibiotic treatment and these variants were able to outcompete the ancestor strain shortly after appearance. Genome sequencing analysis revealed that the adapted *P. aeruginosa* hyperbiofilm variants in different linages shared single-point mutations in the same gene, which encodes the sigma factor RpoS.

The *rpoS* gene has been previously well characterized in *P. aeruginosa* for its regulatory role on quorum sensing and virulence. A DNA microarray-based transcriptomic study showed that the expression of *rpoS* in *P. aeruginosa* biofilm cells was downregulated compared to the planktonic cells, inactivation of *rpoS* in *P. aeruginosa* PAO1 increased biofilm formation in flow-cell reactor [14]. In our study, we demonstrated that *rpoS* point mutations have similar impact to the *rpoS* deletion on biofilm formation in *P. aeruginosa*. Function domain analysis indicates that RpoS contain 4 regions, RpoS as a global regulator, the DNA binding domain was located on region 4. Proline residues are restricts to the first four positions of an α-helix [55], which plays a special role in the stable of protein structure. RpoS^P251L^ and RpoS^Q266stop^ on *P. aeruginosa* genome have shown the same phenotype of hyperbiofilm, pyocyanin production and virulence as Δ*rpoS* strain, which means the 251proline to leucine mutation in *rpoS* might results loss of the function on regulation. Moreover, we showed that mutation in *rpoS* increased the production of c-di-GMP and pyocyanin, both of those two molecules were play a very important role in biofilm formation [39] and virulence [56] in *P. aeruginosa*.

It is generally accepted that bacterial cells employ biofilm mode of growth during chronic infections with reduced level of virulence. For example, the alginate-over producing *P. aeruginosa* Δ*mucA* mutant and the high c-di-GMP containing Δ*wspF* mutant both produce less amounts of virulence factors compared to the wild-type strains [57, 58]. However, here we showed that *rpoS* mutation enable enhancement of both biofilm formation and production of virulence factors such as pyocyanin. The reactive oxygen species generated by redox cycling of pyocyanin kills host and pathogen cells, resulting in extracellular DNA (eDNA) release [59, 60]. eDNA has been shown plays a central role in biofilm formation by increasing biofilm integrity and strength [61–63]. Not limited to the *P. aeruginosa* species, *rpoS* mutants were also reported to contribute to population heterogeneity in *Escherichia coli* O157:H7 strains [64].

Biofilm formation as a response to ecological competition [65], PAO1 wild-type and *rpoS* mutants live with identical niches and also have identical needs within biofilm, thus they will compete for precisely the same resources. Our observation that *rpoS* mutants can outcompete the PAO1 within biofilms could be explained by the competitive exclusion principle [66, 67]. The *P. aeruginosa* small colony variants (e.g., with *wspF* mutations) are also well known being evolved in *P. aeruginosa* biofilms, which have even higher biofilm formation capacity than the *rpoS* mutants. However, these small colony variants often have a lower planktonic growth rate than the *P. aeruginosa* wild-type strain, and thus can easily be outcompeted by the wild-type in the planktonic phase of growth [27]. Instead, our study showed that the *P. aeruginosa rpoS* mutants are able to outcompete the wild-type PAO1 strain within biofilm while not compromising its planktonic growth rate. MIC measurement experiments showed no antimicrobial resistance difference between the *rpoS* mutants and PAO1, suggesting a form of antimicrobial tolerance of biofilm cells. Antimicrobial tolerance is one of the most important features of microbial biofilms, which does not involve drug resistance mutations [68]. The main mechanism of antimicrobial tolerance of biofilms is the reduction of antibiotic penetration [69]. Our study here showed that rapid selection of the hyperbiofilm variants carrying the *rpoS* mutations represents a novel antimicrobial tolerance mechanism of biofilms.

## Conclusions

Our study showed that imipenem treatment drives rapid evolution of *P. aeruginosa rpoS* deficient mutants within biofilms. We provided evidence that *rpoS* mutation not only increase *P. aeruginosa* virulence, but also enhance its intracellular c-di-GMP content. Importantly, the major obstacle for treatment of *P. aeruginosa* infection in clinical is the formation of biofilms. As a sigma factor, RpoS controls a wide range of genes under stationary phase of growth, which shared many characters with biofilm mode of growth, such as lack of nutrients and accumulation of waste products. Further studies should be carried to examining the regulatory roles of RpoS on *P. aeruginosa* physiology and virulence factors under biofilm mode of growth. This study raises the possibility that some clinical *P. aeruginosa* strains with *rpoS* mutations could have a selective advantage during imipenem administration, which might have an impact on the antibiotic therapy against *P. aeruginosa* biofilm-associated infections.

## Methods

For details see Additional file 1.

### Biofilm experimental evolution

The experimental evolution of *P. aeruginosa* PAO1 biofilm was carried out on glass beads formed biofilm [31]. Two autoclaved 5 mm glass beads (Merck KGaA, Darmstadt, Germany) were placed into each well of a 24-well microtiter plate (Jet Biofil, Guangzhou, China). A LB overnight culture of *P. aeruginosa* was diluted in LB to approx. 1 × 10^6^ bacteria per mL and dispensed into the bead-containing 24 well microplate (1 mL per well). The microplate was then placed in a moisture box and incubated at 37 °C for 24 h at 100 rpm on an orbital shaker. After 24 h, the liquid culture was removed, and beads were washed by 0.9% NaCl for twice to remove loosely attached bacteria. Then one bead was transferred into a 2 mL microcentrifuge tube containing 1 mL 0.9% NaCl, subjected to 6 × 10 s vortex and sonicated in an ultrasonic bath (Worldvicon, Shenzhen, China) at 40 kHz for 5 min. Bacterial suspensions were subsequently serially diluted in 0.9% NaCl before being drop-plated onto lysogeny broth agar plates (Difco). After 24 h of incubation at 37 °C, the residual biofilm was quantified as CFU/bead. Another bead was transferring to a 24 well microplate contain 1 mL LB with 160 μg/mL imipenem. The microplate was then placed in a moisture box and incubated at 37 °C for 24 h without shaking. After 24 h treatment, this bead was washed by 0.9% NaCl for twice and transfer into 2 mL microcentrifuge tube containing 1 mL LB, after vortex and sonicated. 20 μL of bacterial suspensions were subsequently serially diluted in 0.9% NaCl before being drop-plated onto lysogeny broth agar plates (Difco), the rest bacterial suspensions were cultured at 37 °C for 24 h at 200 rpm. After 24 h of cultivation, 100 μL *P. aeruginosa* was diluted in LB to approx. 1 × 10^6^ bacteria per mL and start a new cycle. The rest culture was glycerol stocked at − 80 °C. The CFU/bead increased over 100-fold compared to the ancestral strain was defined as hyperbiofilm phenotype variants.

### Biofilm competition assay

The biofilm competition assay was carried out on glass beads formed biofilm. The ancestor strain PAO1 and mutants were tagged with *gfp* and *mcherry* at the *attB* site to generate the strain PAO1 *attB*::*gfp* and mutant *attB*::*mcherry* as previously described [70]. Overnight cultures were adjusted OD_600_ to 1.0, cells were mixed 1:1 or 1:5 and confirmed by flow cytometer analysis. The mixed bacteria were diluted in LB to approx.1 × 10^6^ of per mL and dispensed into the bead-containing 24 well microplate (1 mL per well). The microplate was then placed in a moisture box and incubated at 37 °C for 24 h at 100 rpm on an orbital shaker. After 24 h treatment, the cells in planktonic and biofilm were analyzed by Beckman Cytoflex S flow cytometer. All samples were assayed with lasers emitting at 488 nm for GFP or 561 nm for mCherry. Fluorescence was collected by 530/30 nm bandpass filter for GFP and 615/20 nm bandpass filter for mCherry. In each run, we measured 100,000 events. Sterile PBS, wild-type (WT) *P. aeruginosa PAO1*, mCherry positive and GFP-positive cells were used to gate the cell populations on flow-cytometry diagrams.

### DNA extraction, sequencing, and SNP analysis

Genomic DNA of the ancestor and evolved bacterial populations were extracted form glycerol stocked by AxyPerp Bacterial Genomic DNA Miniprep Kit (Corning) and sequenced by Illumina NovaSeq platform. Illumina genomic reads of the isolates were analyzed by CLC Genomics Workbench 20 (Qiagen) using Resequencing analysis module with default parameters for single nucleotide polymorphism (SNP) with *P. aeruginosa* PAO1 as reference genome.

### RNA extraction, sequencing, and transcriptomic analysis

Samples were collected at the peak of P_*cdrA*_-*gfp* fluorescence intensity, RNA extraction was performed using the miRNeasy kit (Qiagen) according to the manufacturer’s instructions. RNA samples were submitted to Guangdong Magigene Biotechnology Co., Ltd. (Guangzhou, China) for ribosomal RNA depletion and sequencing. RNA samples were sequenced on an Illumina Hiseq Xten platform and 150 bp paired-end reads were generated.

The quality of raw sequence data was assessed using FastQC (Babraham Bioinformatics). Adaptor sequences were removed by adaptor trimming function in CLC. RNA sequence analysis was done using “RNA-seq analysis’ module in CLC genomics Workbench 20 (CLC Bio, Aarhus, Denmark) using *P. aeruginosa* PAO1 reference genome downloaded from NCBI database. Adaptor sequences were removed by adaptor trimming function in CLC. Differential gene expression was analyzed using DESeq2 package in R software.

### Statistical analysis

Data are presented as mean ± standard deviation (SD). All other comparisons were made using a one-way analysis of variance (ANOVA) with Student’s *t* test. Analyses were performed using GraphPad Prism v.7 (GraphPad Software). Statistical significance was determined using a *P* value of < 0.05.

## Supplementary Information

**Additional file 1.** Supplementary methods, figures and tables for this manuscript.

## Data Availability

The DNA and RNA sequence data that support the findings of this study have been deposited in the NCBI Sequence Read Archive (SRA) with the accession number: PRJNA678555. The materials that support the findings of this study are available from the corresponding author upon reasonable request.

## Abbreviations

CF: Cystic fibrosis
CFU: Colony forming units
MIC: Minimum inhibitory concentration
CV: Crystal violet
QS: Quorum-sensing
DGC: Diguanylate cyclases
PDE: Phosphodiesterases
LDH: Lactate dehydrogenase
