# Supplementary material for: rpoS-mutation variants are selected in Pseudomonas aeruginosa biofilms under imipenem pressure
Source: Cell Biosci. 2021 Jul 21;11:138. doi: 10.1186/s13578-021-00655-9 (PMC8293535; doi:10.1186/s13578-021-00655-9)
Supplement: Supplementary file 1 — Additional file 1. Supplementary methods, figures and tables for this manuscript. [file 13578_2021_655_MOESM1_ESM.docx]

**Supplementary Information**

***rpoS*-mutation variants are selected in *Pseudomonas aeruginosa* biofilms under imipenem pressure**

Xiangke Duan^1,2^, Yanrong Pan^2^, Zhao Cai^2^, Yumei Liu^2^, Yingdan Zhang^2^, Moxiao Liu^2^, Yang Liu^3^, Ke Wang^4^, Lianhui Zhang^1^*, Liang Yang^2, 5^*

^1^ Guangdong Province Key Laboratory of Microbial Signals and Disease Control, Integrative Microbiology Research Center, South China Agricultural University, Guangzhou 510642, Guangdong, P. R. China

^2^ School of Medicine, Southern University of Science and Technology, Shenzhen 518055, Guangdong, P. R. China

^3^ Southern University of Science and Technology Hospital, Shenzhen 518055, Guangdong, P. R. China

^4^ Department of Pulmonary and Critical Care Medicine, the First Affiliated Hospital of Guangxi Medical University. Nanning 530021, Guangxi, P. R. China

^5^ Shenzhen Key Laboratory for Gene Regulation and Systems Biology, Southern University of Science and Technology, Shenzhen 518055, Guangdong, P. R. China

*For correspondence: Yangl@sustech.edu.cn, Lhzhang01@scau.edu.cn

Running title: RpoS increases the biofilm formation

**Supplementary Methods**

**Bacterial strains and culture conditions**

The bacterial strains and plasmids used in this study are listed in supplementary Table S2. The clinical isolates were obtained from the first affiliated hospital of Guangxi medical university (ethical approval number [2017(KY-E-080)]. *P. aeruginosa* strain and derivatives were grown at 37 ℃ in LB or ABTGC (ABT minimal medium [1] supplemented with 2 g/L glucose and 2 g/L casamino acids) medium. Antibiotics were used at the following concentrations: for *Escherichia coli*, gentamicin at 30 μg/mL and ampicillin at 100 μg/mL; for *P. aeruginosa*, gentamicin at 60 μg/mL and carbenicillin at 200 μg/mL.

**Biofilm quantification assays**

Microtiter crystal violet (CV) biofilm assays were performed as previously described [2] with some adaptations. Briefly, bacteria are grown overnight at 37 ℃ with shaking, then adjust OD_600_=1.0 and dilute the cultures 1:100 into fresh LB medium. Aliquot 100 μL of diluted cultures were transferred into a 96-well round-bottom polyvinyl chloride (PVC) plate (Corning) and incubated for 16 h at 37 ℃ in a moisture box. Biofilm formed by *P. aeruginosa* strains was stained with 0.1% crystal violet (CV) and the stained CV was dissolved in 30 % acetic acid for measurement of the absorbance at 550 nm.

**Disk diffusion assay**

Overnight cultures were plated on LB agar plates at approx. 10^6^ CFU/mL and left to dry for 15 min before placing a 10 µg/mL imipenem disk (Liofilchem) in the middle of the plate. MIC comparison was done by comparing the radius zone of inhibition.

**Growth rate measurements**

The growth rate of the evolved strains and of the ancestral strain were measured by monitoring OD_600_ in 24-well plates with the multiwell reader Spark (Tecan, Switzerland) at 37 ℃ with shaking.

**Biofilm growth curve**

The biofilm growth curve measurement was carried out on glass beads formed biofilm. Overnight cultures of PAO1, Δ*rpoS* and the complementation strain Δ*rpoS/*p*-rpoS* were diluted in LB to approx. 1x10^6^ bacteria per mL and dispensed into the bead-containing 24 well microplate (1 mL per well). The microplate was then placed in a moisture box and incubated at 37 ℃ for 24 h at 100 rpm on an orbital shaker. At the set time point, bead biofilm and planktonic cultures were collected, serially diluted and plated on LB agar plates for CFU count. Experiments were performed with four replicates, and the results are shown as the mean ± s.d.

**Construction of transcriptional fusions reporter strains**

For the *lasB* [3], *pqsA* [4], *rhlA* [5], *cdrA* [6], *rsmY* and *rsmZ* [7] promoter-reporter fusion strains, the corresponding plasmid was introduced into ancestor and mutants by electroporation. Transformants were selected on LB agar plates containing carbenicillin.

**Transcriptional expression of reporter assay**

Overnight cultures were adjusted OD_600_ to 1.0 and dilute the cultures 1:100 into fresh ABTGC medium. One hundred microliters were transferred to a polystyrene black 96-well plate (Corning). Cells were measured on a Spark plate reader (Tecan) for OD_600_ and GFP ﬂuorescence. Fluorescence was measured as ﬂuorescence intensity units (FIU) using an excitation of 488 nm and emission of 535 nm. The FIU values were normalized to OD, and the average autoﬂuorescence from the empty vector control was subtracted for each strain. Three biological replicates, each with two technical replicates, were performed.

**Targeted resequencing of *rpoS***

*rpoS* was PCR ampliﬁed from the *P. aeruginosa* genome using primers rpoSF and rpoSR (Supplementary Table S3). Fragments were sequenced in both directions with the Sanger method. Sequence analysis was done with Snapgene (GSL Biotech LLC., CA, USA).

**Mutant strain creation through allelic exchange**

The in-frame deletion and site-directed mutagenesis of *rpoS* was performed through two-step allelic exchange [8]. For *rpoS* in-frame deletion, the upstream and downstream DNA fragments of *rpoS* were amplified with two pairs of primers rpoSF1/rpoSR1 and rpoSF2/rpoSR2, respectively. The two PCR products purified by HiPure PCR pure mini kit (Magen) and ligated to *Hind* III and *Eco*R I double digested suicide vector PK18 by Gibson Assembly master mix (NEB) to yield PK18-rpoS. After sequence confirmation, the suicide plasmid PK18-rpoS was mobilized from *E. coli* Top10 (donor strain) to *P. aeruginosa* PAO1 (receptor strain) by conjugal mating with the help of pRK600 vector and selection for gentamycin-resistant first homologous recombinants. Colonies were then streaked on LB agar plates with 15% sucrose to select second homologous recombinants. And the *rpoS* deletion mutant was identified by PCR using primers comrpoSF and s comrpoSR.

For site-directed mutagenesis, the fragments for *rpoS* G752A and G796A substitutions were amplified from C6W1C and C6W6F genome using primers mtrpoSF and mtrpoSR. The PCR products purified and ligated to *Hind* III and *Eco*R I double digested suicide vector PK18 by Gibson Assembly master mix (NEB) to yield suicide plasmid PK18-rpoS_G752A_ and PK18-rpoS_G752A_. The suicide plasmid was mobilized from *E. coli* SM10 λ*pir* (donor strain) to *P. aeruginosa* PAO1 (receptor strain) by conjugal mating and selection for gentamycin-resistant and sucrose-resistant. All resultant mutants were verified by PCR and DNA sequencing.

**Complementation of *rpoS* mutant strains**

Wild-type PAO1 *rpoS* with its promoter region [9] was PCR ampliﬁed from PAO1 genomic DNA, the PCR product was purified and ligated to *Afl* II and *Hind* III double digested pHERD20T [10] by Gibson Assembly master mix (NEB) to yield p20T-PrpoS. After sequencing, the plasmids were transformed into competent Δ*rpoS* or evolved mutants by electroporation. The positive colonies grown on LB plates supplemented with carbenicillin were confirmed by plasmid isolation and restriction enzyme digestion analysis.

**Screening for high biofilm clinical strains**

To determine the appearance of *rpoS* mutations in clinical *P. aeruginosa* strains and its association with hyperbiofilm phenotype, we screened 288 isolates for biofilm production by microtiter crystal violet biofilm assay. The clinical *P. aeruginosa* isolates were obtained from the First Affiliated Hospital of Guangxi medical university. Single colony was picked into sterile 96-well microtiter plates containing 100 µL of LB media. Plates were incubated statically for 24 hours at 800 rpm at 37°C until the bacteria reached the stationary phase of growth. Dilute the stationary phase cultures 1:100 into fresh LB medium and aliquot 100 μL of diluted cultures were used for biofilm assay. Because the capacity to form biofilm is proportional to the bacterial cell growth, OD_550_ nm was normalized with cell mass determined by measuring OD_600_ nm. The experiment was performed in duplicate.

**Pyocyanin production**

Pyocyanin production measurement was performed as described previously [11]. Brieﬂy, Overnight cultures were diluted to an OD_600_ of 1.0, and 50 μL was used to inoculate 5 ml of LB. After 18 h, the cultures were centrifuged, and the resultant supernatant was then used for pyocyanin quantiﬁcation. In brief, 3 mL of chloroform was added to 5 mL of supernatant, and the tube was vortexed twice for 10 s. The tubes were centrifuged at 7,000 rpm for 10 min, and the bottom phase was transferred to a new tube containing 1 mL of 0.2 N HCl. The tubes were vortexed twice for 10 s each time and centrifuged at 7,000 rpm for 2 min. The OD_520_ of the top phase was measured.

**Cytotoxicity assay**

The cytotoxicity of the ancestral, mutation and clinical isolate strains were assayed by using murine RAW 264.7. RAW macrophages were grown in 24-well plates in Dulbecco modiﬁed Eagle medium (DMEM), GlutaMAX, sodium pyruvate, and phenol red supplemented with 10% FBS. Prior to infection, conﬂuent RAW cells were washed twice with sterile PBS and incubated in DMEM medium. The log-phase cultures washed with sterile PBS twice, and resuspended in DMEM medium devoid of FBS. Macrophages cells were infected with bacteria at a Multiplicity of Infection (MOI) of 20 at 37 ℃ in 5% CO_2_ incubator. After 4 h infection, the culture supernatants were collected for detecting lactate dehydrogenase (LDH) activities. The LDH activities were detected by using commercially LDH cytotoxicity kit (YAESEN Bio) according to standard procedure.

**Supplementary Figures**

**
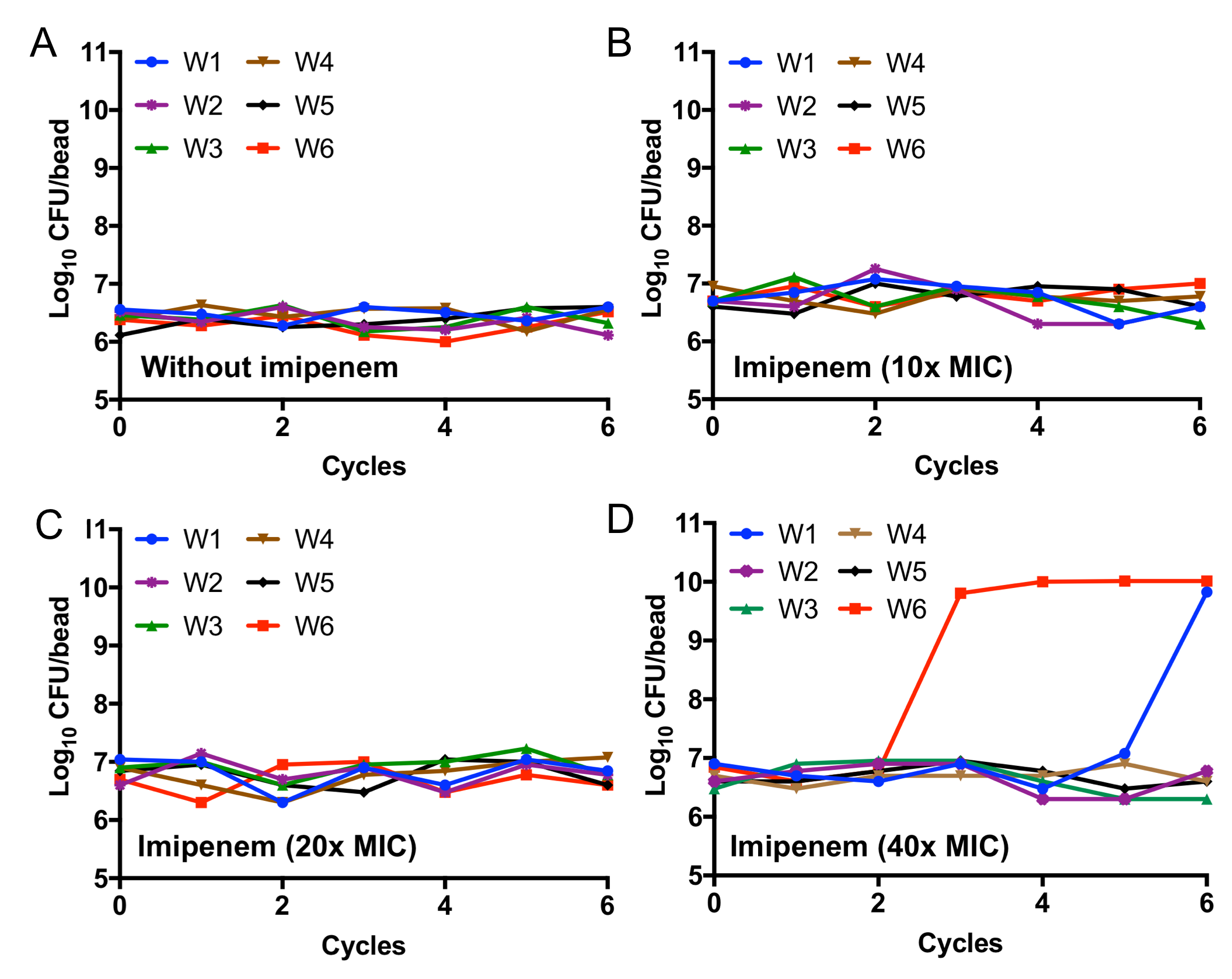
**

**Figure S1. Hyperbiofilm variants were accumulated upon 40****x MIC of imipenem treatment.** The CFU of biofilm bacteria on bead in control group (A), 10 (B), 20 (C) and 40x MIC (D) imipenem treated group were measured at each cycle.


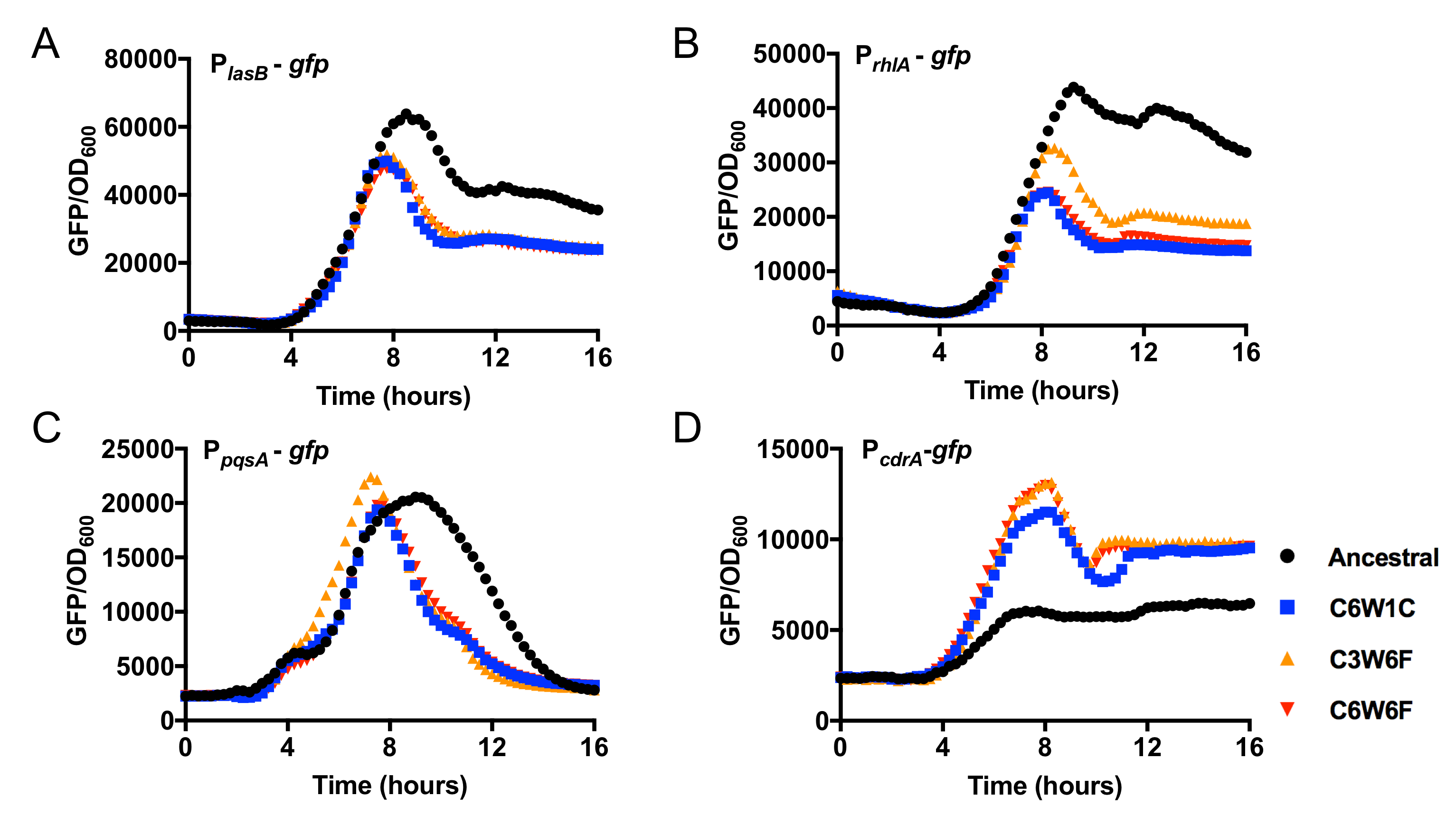


**Figure S2. The measurement of quorum-sensing and c-di-GMP levels by transcriptional fusion reporters.** The strains harbor fluorescence reporters for las system (A), rhl system (B), pqs system (C) and c-di-GMP level (D) were incubated in 96 wells plate at 37 ℃ in Spark plate reader (Tecan), OD_600_ and GFP ﬂuorescence were continuous measured for 16 h. The GFP/OD_600_ value was reflect the expression level of corresponding systems in representative strains. Data are presented as the mean±s.d. of five biological replicates.


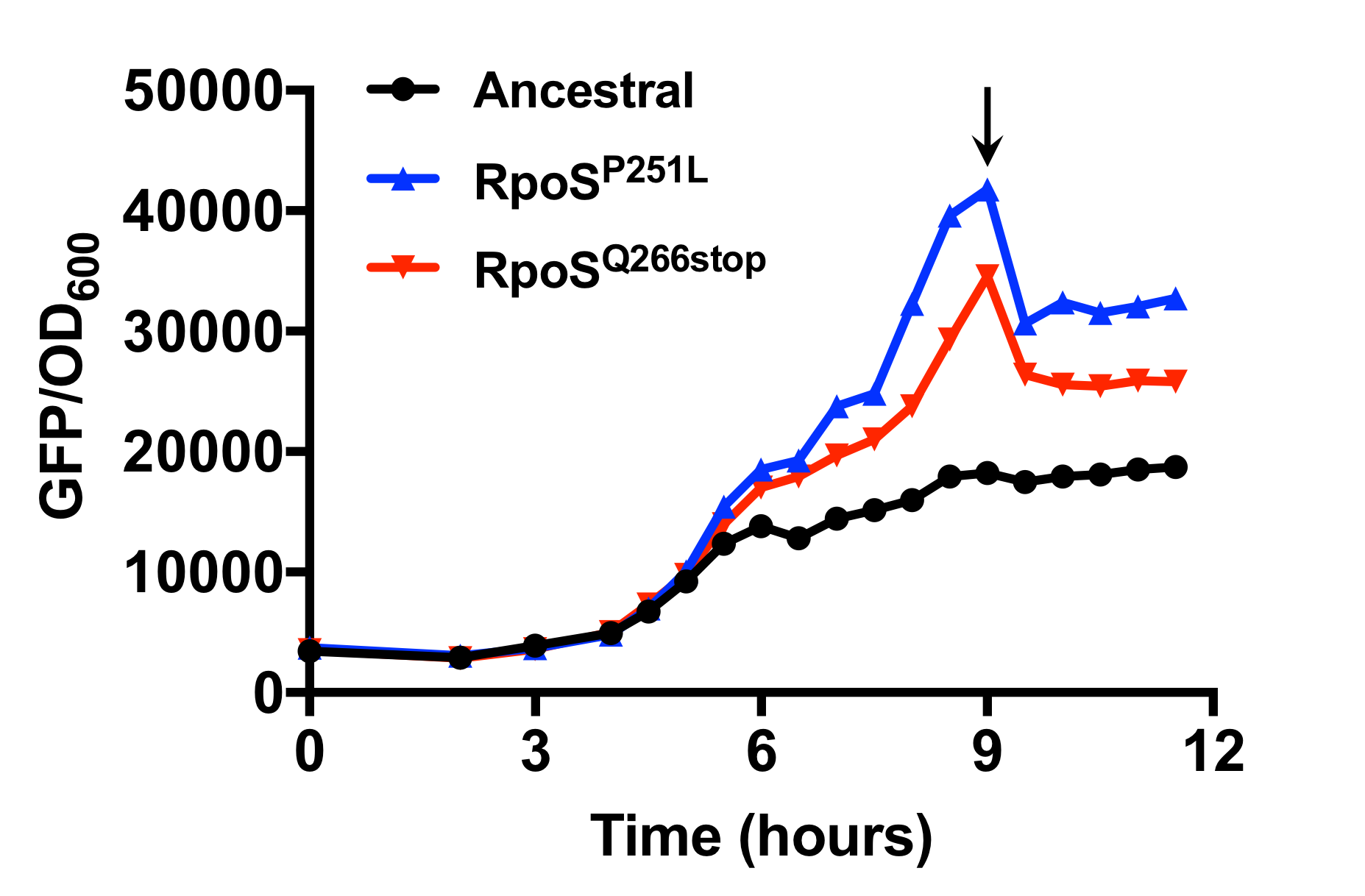


**Figure S3. The time point selected to collect bacterial cultures for RNA-seq.** Strains were cultured in 24 wells plate at 37 ℃ in ABTGC, at designed time point, OD_600_ and GFP ﬂuorescence were measured. Samples were collected at the peak of GFP/OD_600_ value. Data are presented as the mean±s.d. Data are presented as the mean±s.d. of six biological replicates.

**Table S1. Isolation information of clinical *P. aeruginosa* strains.**

| Isolation site | Number of isolates | Hyperbiofilm isolate |
| --- | --- | --- |
| Sputum | 171 | 18 |
| Blood | 12 | 3 |
| Burn wound | 35 | 3 |
| Urine | 14 | 1 |
| Femoral vein indwelling tube | 2 | 1 |
| Peritoneal drainage fluid | 3 | 1 |
| Bile | 4 | 1 |
| Drawing fluid | 1 | 0 |
| Throat swab | 1 | 0 |
| secretions of surgical wounds | 8 | 0 |
| Central venous catheters | 2 | 0 |
| Wound secretion | 1 | 0 |
| Wound | 4 | 0 |
| Cut wound | 2 | 0 |
| Tracheostomy tube | 1 | 0 |
| Tracheal intubation | 1 | 0 |
| Airway secretion | 1 | 0 |
| Pelvic drainage tube | 1 | 0 |
| Pus | 1 | 0 |
| Purulent secretion | 2 | 0 |
| Ulcer secretion | 1 | 0 |
| Mouth Sore Swab | 1 | 0 |
| Necrotic tissue | 1 | 0 |
| Ascitic fluid | 2 | 0 |
| T tube drainage fluid | 1 | 0 |
| Bronchoalveolar Lavage Fluid | 7 | 0 |
| Other | 2 | 0 |

**Table S2. Bacterial strains and plasmids in this study.**

|  | Description ^a^ | Source of ref. |
| --- | --- | --- |
| **Plasmids** |  |  |
| pK18 | Small mobilizable vector, Gm^R^, sucrose sensitive (sacB) | [12] |
| RK600 | Cm^R^, ColE1, oriV, RK2, mob^+^, tra^+^; helper plasmid in triparental matings | [13] |
| pBF13 | R6K replicon-based helper plasmid, providing the Tn7 transposition function in *trans*; Amp^R^, *mob*+ | [14] |
| pHERD20T | Empty vector; Carb^R^ | [10] |
| Mini Tn7-*mcherry* | Gm^R^ on mini-Tn7T; mobilizable; for mCherry tagging | [15] |
| Mini Tn7-*gfp*3 | Gm^R^ on mini-Tn7T; mobilizable; for GFP tagging | [16] |
| pUCP22::*lasB*-*gfp* | *lasB* promoter fused to *gfp*; Carb^R^ | [3] |
| pUCP22::*pqsA*-*gfp* | *pqsA* promoter fused to *gfp*; Carb^R^ | [4] |
| pUCP22::*rhlA*-*gfp* | *rhlA* promoter fused to *gfp*; Carb^R^ | [5] |
| pUCP22::*cdrA*-*gfp* | *cdrA* promoter fused to *gfp*; Carb^R^ | [6] |
| pUCP22::*rsmY*-*gfp* mut3 | *rsmY* promoter fused to *gfp* mut3; Carb^R^ | [7] |
| pUCP22::*rsmZ*-*gfp* mut3 | *rsmZ* promoter fused to *gfp* mut3; Carb^R^ | [7] |
| **Strains** |  |  |
| ***E. coli*** |  |  |
| TOP10 | F-, *mcrA* Δ(*mrr*-*hsd* RMS-*mcr* *BC*) *φ80lacZ* Δ*M15* Δ*lac* *X74* *recA1* *ara* Δ*139* Δ(*ara*-l*eu*) *7697* *galU* *galK* *rpsL* (Strr) *endA1* *nupG* | TIANGEN |
| ***P. aeruginosa strains*** | | |
| PAO1 | Wild type | This lab |
| C6W1C | Hyperbiofilm evolution strain of PAO1 | This study |
| C3W6F | Hyperbiofilm evolution strain of PAO1 | This study |
| C6W6F | Hyperbiofilm evolution strain of PAO1 | This study |
| Δ*rpoS* | *rpoS* deletion mutant of PAO1 | This study |
| RpoS^P251L^ | RpoS^P251L^ mutation of PAO1 | This study |
| RpoS^Q66stop^ | RpoS^Q66stop^ mutation of PAO1 | This study |
| #16 | Clinical isolated hyperbiofilm *P. aeruginosa* strain | This study |
| *rpoS*ΔR3 | *rpoS* region 3 deletion mutant of PAO1 | This study |
| *rpoS*ΔR4 | *rpoS* region 4 deletion mutant of PAO1 | This study |
| *rpoS*ΔR34 | *rpoS* region 3 and 4 deletion mutant of PAO1 | This study |
| *rpoS*ΔR234 | *rpoS* region 2, 3 and 4 deletion mutant of PAO1 | This study |
| *rpoS*ΔR1 | *rpoS* region 1 deletion mutant of PAO1 | This study |
|  |  |  |

a. The following abbreviations are used: Gm ^R^, gentamycin resistance; Cm ^R^, chloramphenicol resistance; Carb^R^, carbenicillin resistance.

**Table S3. Primes used in this study.**

| Name | Sequence (5’-3’) | Application |
| --- | --- | --- |
| rpoSF | CACATCATGTAGGTGAGCGGGTC | For *rpoS* target sequence |
| rpoSR | CGTGTAAGTTAATGCTTACAAGA | For *rpoS* target sequence |
| rpoSF1 | AGCTCGGTACCCGGGACTCGGTACGTTTCGTCCCGCTGCT | For *rpoS* deletion |
| rpoSR1 | CTTCAGTGGGTCTAAGGTTTTCCGGTCGTTATCCCTTGCATGAGTTCG | For *rpoS* deletion |
| rpoSF2 | CGAACTCATGCAAGGGATAACGACCGGAAAACCTTAGACCCACTGAAG | For *rpoS* deletion |
| rpoSR2 | CGACGGCCAGTGCCACTGGCGGAAGTCTGGCCGAACATCA | For *rpoS* deletion |
| comrpoSF | GGCATTTATCTACTTAGGCTCACA | For the confirmation of *rpoS* deletion |
| comrpoSF | TAAGCCTGTCGATCCACTGCAAT | For the confirmation of *rpoS* deletion |
| 20TrposF | CCATGGGATCTGATAAGAATTCATGGCACTCAAAAAAGAAGGGCC | For *rpoS* complement |
| 20TrposR | AACGACGGCCAGTGCCAAGCTTTCACTGGAACAGCGCGTCACTCG | For *rpoS* complement |
| mtrpoSF | AGCTCGGTACCCGGGCTTCCTTCTCTTCCAAACAACA | For rpoS P251L and Q266stop mutation |
| mtrpoSR | CGACGGCCAGTGCCAATACGCCAAGGCCCGCAATCA | For rpoS P251L and Q266stop mutation |
|  |  |  |
| R1koF1 | AGCTCGGTACCCGGGACGCTGCTTGTCGGTGAGTTCCGTC | For *rpoS* region 1 deletion |
| R1koR1 | CACATCGACTACACGCGCGCGTTGGGTCGGAAGCGGATGATCGAGAGC | For *rpoS* region 1 deletion |
| R1koF2 | GCTCTCGATCATCCGCTTCCGACCCAACGCGCGCGTGTAGTCGATGTG | For *rpoS* region 1 deletion |
| R1koR2 | CGACGGCCAGTGCCATCTGTTCCCTTCTCGCCGCCTGTTC | For *rpoS* region 1 deletion |
| R3koF1 | AGCTCGGTACCCGGGACTCTTCAGTCCCTCGTCATCAT | For *rpoS* region 3 deletion |
| R3koR1 | CGCTTGCCGATCCATGTGGTCAAGCAGGATGACGATCTCAGCGAAAGC | For *rpoS* region 3 deletion |
| R3koF2 | GCTTTCGCTGAGATCGTCATCCTGCTTGACCACATGGATCGGCAAGCG | For *rpoS* region 3 deletion |
| R3koR2 | CGACGGCCAGTGCCAAGTTAGTACGTCGGTACCTGCCAA | For *rpoS* region 3 deletion |
| R4koF1 | AGCTCGGTACCCGGGAACATCACCGAGAAGAAGGATGCC | For *rpoS* region 4 deletion |
| R4koR1 | GATCTCAGCGAAAGCATCGACCAGATTCTGGAGAAGAATGGCCTGTCG | For *rpoS* region 4 deletion |
| R4koF2 | CGACAGGCCATTCTTCTCCAGAATCTGGTCGATGCTTTCGCTGAGATC | For *rpoS* region 4 deletion |
| R4koR2 | CGACGGCCAGTGCCATTGGTCATCATCAAACACAACGAG | For *rpoS* region 4 deletion |
| R34koF1 | AGCTCGGTACCCGGGAGAACATGCAGGAATTCATCGAGCT | For *rpoS* region 3 and 4 deletion |
| R34koR1 | GCCATCATGAACCAGACCCGGACCTGACGGAAAACCTTAGACCCACTG | For *rpoS* region 3 and 4 deletion |
| R34koF2 | CAGTGGGTCTAAGGTTTTCCGTCAGGTCCGGGTCTGGTTCATGATGGC | For *rpoS* region 3 and 4 deletion |
| R34koR2 | CGACGGCCAGTGCCACAGTTAGTACGTCGGTACCTGCCAA | For *rpoS* region 3 and 4 deletion |
| R234koF1 | AGCTCGGTACCCGGGAGAACATGCAGGAATTCATCGAGCT | For *rpoS* region 2, 3 and 4 deletion |
| R234koR1 | CTGGCGCAGAAGGGCGATCCCGCTTGACGGAAAACCTTAGACCCACTG | For *rpoS* region 2, 3 and 4 deletion |
| R234koF2 | CAGTGGGTCTAAGGTTTTCCGTCAAGCGGGATCGCCCTTCTGCGCCAG | For *rpoS* region 2, 3 and 4 deletion |
| R234koR2 | CGACGGCCAGTGCCAGCGACACCCTGTATTCCATTGCCTT | For *rpoS* region 2, 3 and 4 deletion |

**References**

1. Chua SL, Tan SY, Rybtke MT, Chen Y, Rice SA, Kjelleberg S, Tolker-Nielsen T, Yang L, Givskov M: Bis-(3'-5')-cyclic dimeric GMP regulates antimicrobial peptide resistance in Pseudomonas aeruginosa. *Antimicrob Agents Chemother* 2013, 57:2066-2075.

2. O'Toole GA: Microtiter dish biofilm formation assay. *J Vis Exp* 2011.

3. Hentzer M, Riedel K, Rasmussen TB, Heydorn A, Andersen JB, Parsek MR, Rice SA, Eberl L, Molin S, Hoiby N, et al: Inhibition of quorum sensing in Pseudomonas aeruginosa biofilm bacteria by a halogenated furanone compound. *Microbiology* 2002, 148:87-102.

4. Yang L, Barken KB, Skindersoe ME, Christensen AB, Givskov M, Tolker-Nielsen T: Effects of iron on DNA release and biofilm development by Pseudomonas aeruginosa. *Microbiology* 2007, 153:1318-1328.

5. Yang L, Rybtke MT, Jakobsen TH, Hentzer M, Bjarnsholt T, Givskov M, Tolker-Nielsen T: Computer-aided identification of recognized drugs as Pseudomonas aeruginosa quorum-sensing inhibitors. *Antimicrobial agents and chemotherapy* 2009, 53:2432-2443.

6. Rybtke MT, Borlee BR, Murakami K, Irie Y, Hentzer M, Nielsen TE, Givskov M, Parsek MR, Tolker-Nielsen T: Fluorescence-based reporter for gauging cyclic di-GMP levels in Pseudomonas aeruginosa. *Applied and environmental microbiology* 2012, 78:5060-5069.

7. Chua SL, Liu Y, Yam JK, Chen Y, Vejborg RM, Tan BG, Kjelleberg S, Tolker-Nielsen T, Givskov M, Yang L: Dispersed cells represent a distinct stage in the transition from bacterial biofilm to planktonic lifestyles. *Nat Commun* 2014, 5:4462.

8. Hmelo LR, Borlee BR, Almblad H, Love ME, Randall TE, Tseng BS, Lin C, Irie Y, Storek KM, Yang JJ, et al: Precision-engineering the Pseudomonas aeruginosa genome with two-step allelic exchange. *Nat Protoc* 2015, 10:1820-1841.

9. Thi Bach Nguyen H, Romero AD, Amman F, Sorger-Domenigg T, Tata M, Sonnleitner E, Blasi U: Negative Control of RpoS Synthesis by the sRNA ReaL in Pseudomonas aeruginosa. *Front Microbiol* 2018, 9:2488.

10. Qiu D, Damron FH, Mima T, Schweizer HP, Hongwei DY: PBAD-based shuttle vectors for functional analysis of toxic and highly regulated genes in Pseudomonas and Burkholderia spp. and other bacteria. *Applied and environmental microbiology* 2008, 74:7422-7426.

11. Essar DW, Eberly L, Hadero A, Crawford I: Identification and characterization of genes for a second anthranilate synthase in Pseudomonas aeruginosa: interchangeability of the two anthranilate synthases and evolutionary implications. *Journal of bacteriology* 1990, 172:884-900.

12. Schäfer A, Tauch A, Jäger W, Kalinowski J, Thierbach G, Pühler A: Small mobilizable multi-purpose cloning vectors derived from the Escherichia coli plasmids pK18 and pK19: selection of defined deletions in the chromosome of Corynebacterium glutamicum. *Gene* 1994, 145.

13. Kessler B, de Lorenzo V, Timmis KN: A general system to integratelacZ fusions into the chromosomes of gram-negative eubacteria: regulation of thePm promoter of theTOL plasmid studied with all controlling elements in monocopy. *Molecular and General Genetics MGG* 1992, 233:293-301.

14. Bao Y, Lies DP, Fu H, Roberts GP: An improved Tn7-based system for the single-copy insertion of cloned genes into chromosomes of gram-negative bacteria. *Gene* 1991, 109:167-168.

15. Lagendijk EL, Validov S, Lamers GE, De Weert S, Bloemberg GV: Genetic tools for tagging Gram-negative bacteria with mCherry for visualization in vitro and in natural habitats, biofilm and pathogenicity studies. *FEMS microbiology letters* 2010, 305:81-90.

16. Choi K-H, Schweizer HP: mini-Tn 7 insertion in bacteria with single att Tn 7 sites: example Pseudomonas aeruginosa. *Nature protocols* 2006, 1:153-161.
